# Ultrasonic Levitation for Airway Humidification

**DOI:** 10.3390/s24144691

**Published:** 2024-07-19

**Authors:** Riaz Uddin, Ahmed M. Al-Jumaily

**Affiliations:** Institute of Biomedical Technologies, Auckland University of Technology, Auckland 1010, New Zealand; riazz99@gmail.com

**Keywords:** ultrasonic transducers, ultrasonic levitator, airway humidification, humidifier, high intensity ultrasound, two stage ultrasonic humidifier

## Abstract

This study employs the transmitter part of an ultrasonic proximity sensor to generate a powerful ultrasonic field for medical humidification. This field is created using an arrangement of small ultrasonic transmitter transducers configured in an acoustic levitator-style setup. As droplets pass through this ultrasonic field, they undergo disintegration, leading to an accelerated evaporation process. The research findings highlight a significant change in droplet size distribution due to ultrasonics, resulting in a notable increase in the rate of evaporation. As a result, this study presents a conceptual framework for reimagining humidification devices for lung therapeutic purposes through the utilization of simple sensor technology.

## 1. Introduction

Humidification plays a crucial role in lung therapeutic devices to alleviate the complications caused by dry air. However, the humidification needs of medical devices differ significantly from those met by currently available ultrasonic humidifiers. Specifically, for breathing devices, humidifiers must be capable of producing vapours consisting of water particles smaller than 1 µm in diameter. The progression of ultrasonic transmitters has led to the exploration of numerous studies aimed at enhancing humidification outcomes through ultrasound. Kurosawa et al. [1] proposed a compact humidifier design called “dry fog generation”, which utilized a surface acoustic wave (SAW) transducer. Their design involved using a 4 × 8 × 0.6 mm^3^ flat plate made of LiNbO_3_ (Lithium Niobate) as a piezoelectric material, along with an interdigital aluminum transducer for generating acoustic waves. The transducer comprised 40 electrodes connected to a Radio Frequency power amplifier, producing a high-frequency electrical power of 48 MHz. Acoustic radiation from the SAW device surface induced capillary waves, causing the disintegration of small liquid particles. The authors claimed to have achieved droplets with a diameter of 5 μm. The atomizing rate was reported as 170 µL/min, with an input power of 2.3 W (36 V). Another design by Yuan et al. [2,3] involved a micromachined nozzle shape and utilized bending mode Lead Zirconate Titanate (PZT) at an operating frequency of 36 kHz (at 70 or 80 V). However, this design fails to meet the humidification requirements for medical devices. Tsai et al. [4] presented a micro-electro-mechanical system (MEMS)-based miniaturized silicon ultrasonic droplet generator, incorporating multiple Fourier horns within a simple nozzle architecture. The experiment involved two different liquids, with water resulting in droplets with a diameter of 4.5 μm and ethanol producing droplets measuring 2.4 μm in diameter. However, due to its operating frequency of 1 to 1.5 MHz, the design does not support continuous production. Another design by Tsai et al. [5,6] was a low-power, pocket-size nebulizing nozzle. The system’s dimensions were 8.8 × 5.9 × 1.9 cm, and it claimed to produce aerosol particles of desirable sizes and an output rate of 0.15 mL/min. It utilized a silicon-based MHz MFHUN (multiple-Fourier horn ultrasonic nozzle) to generate monodisperse droplets within the desired size range of 2 to 5 μm. The system demonstrated moderate output (up to 0.2 mL/min) at low electrical drive power (sub-Watt). Although the design allowed for multiple nozzles to be used in parallel, enabling a high output rate, it still did not achieve continuous vaporization. Soluch and Wrobel [7] described a low-power SAW atomizer. Their design utilized a unidirectional interdigital transducer, a horn, and a waveguide fabricated on a LiNbO_3_ substrate. Operating at a frequency of 78 MHz with a power of 1 W, the device achieved continuous atomization of water. The droplet diameter reached up to 1.5 µm, and the atomizing rate was approximately 40 µL/min, which is close to medical humidification requirements. However, the repeatability and uniformity of the atomization process were not deemed reliable.

A study conducted by Protheroe [8] drew inspiration from a design involving a cylindrical chamber driven by a piezoelectric transducer, which was used for drying foodstuff with an acoustic field inside the transducers [9,10]. Protheroe’s study aimed to experiment with a similar concept for human airway humidification, utilizing an atomizer to generate droplets and a comparable type of chamber for evaporating those droplets. In Protheroe’s setup, a cylindrical sonotrode was attached to an ultrasonic transducer, requiring 75 W of input power to generate an acoustic output of 155 dB for evaporation. Another study conducted by Meshkinzar and Al-Jumaily [11,12] further advanced the approach of human airway humidification using a cylindrical piezoelectric element with stepped thickness to enhance focus. This study improved the ultrasound’s focus using stepped thickness, thereby increasing intensity and enhancing evaporation results. Consequently, the implementation of a 2-stage ultrasound approach, as opposed to single ultrasound vaporization, has demonstrated superior output in terms of humidification.

To further enhance droplet vaporization, high-intensity ultrasound can be used at the second stage, which further disintegrates droplets and ultrasonic force impact. The concept of a high-intensity ultrasound levitator setup is investigated. Previous studies have demonstrated the effective disintegration of liquid droplets using high-intensity ultrasound [13]. The disintegration of droplets generates smaller droplets in the air. The decrease in droplet size increases the overall vaporization rate, and can be understood theoretically from Kelvin’s equation, which states that the rate of evaporation is inversely proportional to the droplet size, indicating that a decrease in droplet size leads to an increase in the evaporation rate [14].

(1) $$ln\frac{P_{vap}^{curved}}{P_{vap}^{flat}}=\frac{2oV_{m}}{rRT}$$

where $P_{vap}^{curved}$ is the vapour pressure on curve edges of droplet, $P_{vap}^{flat}$ is the vapour pressure on flat surface, o is the surface tension, r is the radius of curvature, ***R*** is the universal gas constant, ***T*** is the Temperature.

The equation derived from Kelvin’s equation, when integrated and applied to droplet and air conditions and combined with the heat conduction equation, can be expressed as follows:

(2) $$\frac{X_{w}P_{w}^{vap}\left(T_{s}\right)e^{\frac{2oV_{m}}{rRT_{s}}}}{P_{w}^{vap}\left(T_{\infty }\right)}=e^{-\frac{M_{w}{\dot{m}}_{T}\lambda^{2}w}{4\pi rk_{TH}RT_{\infty }T_{s}}}$$

where $x_{w}$ is the mole fraction of water in the droplet, $P_{w}^{vap}$(*Ts*) is the vapour pressure of water at temperature ***Ts***, $M_{w}$ is the molecular weight of water,$\;{\dot{m}}_{T}$ the evaporation rate of water due to thermal conduction, $T_{\infty }$ is the temperature of surrounding humid air (K), *k*_*TH*_ is thermal conductivity (W/m·K) and ***λ_w_*** is the latent heat of vaporization.

The equation can be rearranged and approximated by considering the first two terms of the exponential function expansion. This approximation yields the evaporation rate due to thermal conduction and the mass lost due to diffusion (****${\dot{m}}_{D})$. Diffusion and heat transfer are the two phenomena contributing to evaporation, expressed as ${\dot{m}}_{T}$ and ${\dot{m}}_{D}$ respectively.

(3) $$\frac{dr}{dt}=\frac{1}{\rho r}\left\{\frac{DM_{w}}{R}\left[\frac{x_{w}P_{w}^{vap}\left(T_{s}\right)e^{\frac{2oV_{m}}{rRT_{s}}}}{T_{s}}-\frac{(RH)P_{w}^{vap}\left(T_{\infty }\right)}{T_{\infty }}\right]+\frac{k_{TH}RT_{\infty }T_{s}}{M_{w}\lambda_{w}^{2}}\left[1-\frac{X_{w}P_{w}^{vap}\left(T_{s}\right)e^{\frac{2oV_{m}}{rRT_{s}}}}{P_{w}^{vap}\left(T_{\infty }\right)}\right]\right\}$$

By simplifying and integrating the equation with the initial condition at *t* = 0, *r* = *r*0, we can express the evaporation rate as a function of the droplet radius. This equation can be solved numerically and the solution placed with sample solution in Appendix A, and when *B* ≪ *A*, the result is:

(4) $$r^{2}-r_{0}^{2}=\frac{2}{\rho }\left\{\frac{DM_{w}}{R}\left[\frac{x_{w}P_{w}^{vap}\left(T_{s}\right)}{T_{s}}-\frac{(RH)P_{w}^{vap}\left(T_{\infty }\right)}{T_{\infty }}\right]+\frac{k_{TH}RT_{\infty }T_{s}}{M_{w}\lambda_{w}^{2}}\left[1-\frac{X_{w}P_{w}^{vap}\left(T_{s}\right)}{P_{w}^{vap}\left(T_{\infty }\right)}\right]\right\}\;t$$

## 2. Materials and Methods

When sinusoidal sound waves are reflected or generated in the opposite direction, they create a standing wave pattern with consistent acoustic pressure at each point and maximum pressure at the nodes. This significant pressure can be harnessed for various applications such as acoustic levitation, holding, and even breaking of droplets [15,16]. Anilkumar et al. [13,16] conducted a study describing various patterns of droplet disintegration in ultrasonic levitation. In the presence of standing ultrasonic waves, droplets experience levitation, and the high sound intensity induces the formation of capillary waves. As the ultrasound pressure is further increased, the droplets can undergo atomization and emit satellite drops due to the instability of the capillary waves.

**Figure 1:**
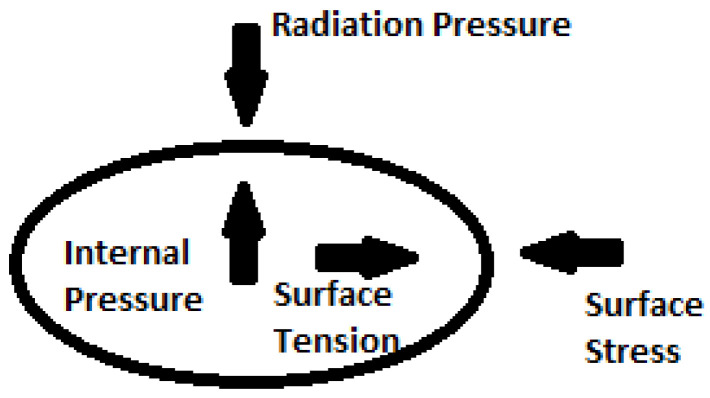
Forces and their directions in a levitated droplet.

The surface tension is caused by intermolecular interactions, which result in the spherical shape of a droplet, while the force exerted on the droplet’s surface is known as surface stress. Before disintegration, the droplets deform, resembling an upward-buckling umbrella, or they expand horizontally while decreasing in thickness until they eventually break down; see Figure 1. The capillary waves observed in the droplets result from the membrane’s vibration induced by the sound pressure. As the membrane’s thickness decreases and the vibration intensifies, atomization occurs. This vibration leads to destabilizing internal pressure, known as the Bernoulli correction.

Earlier studies focused on a single droplet, which cannot be favourable for continuous vaporization. This study focuses on continuous flow investigation when passed over the high-intensity ultrasound.

A 2-stage vaporization setup has been designed for the study. In the first stage, droplets are generated through a nebulizer, and in the second stage, these droplets pass over the levitator setup. Previous studies [17,18,19] have employed high-power acoustic transmitters with reflectors to achieve the desired outcomes. However, using high-intensity power in the patient circuit is not suitable, so for this study, the preference is to utilize compact, highly efficient off-the-shelf transducers. Marzo [20,21,22] employed a multi-transducer array for the levitation and manipulation of particles in the air. In this study, a small compact transducer has been selected and utilized for the generation of the ultrasonic field. To complete the design of the levitator, simulations and circuit design for the transducer have been carried out.

### 2.1. Modelling and Simulations

**Figure 2:**
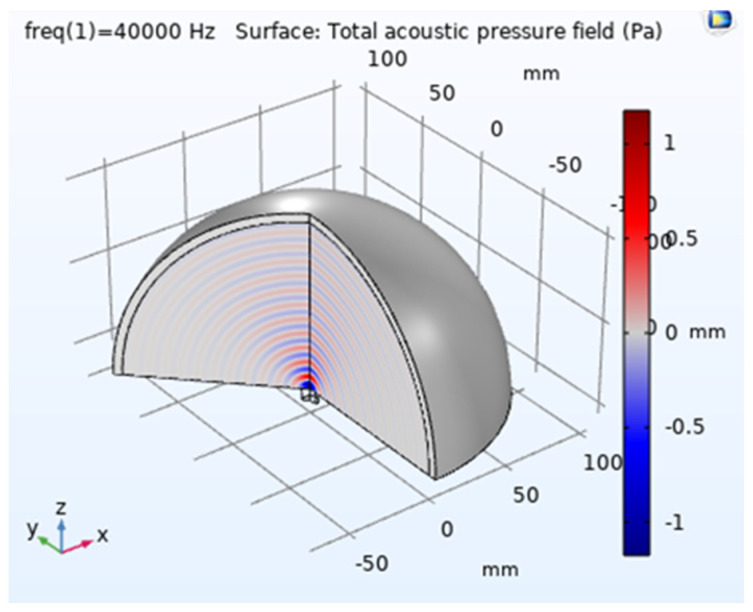
3D presentation of the acoustic waveform from the transducer. Red lines represent the crests and blue lines represent the troughs. The maximum total acoustic pressure at any point on the system was found to be 1.2 Pa, and the maximum pressure point was close to the transducer.

In COMSOL Version 5.6. simulation, a 2D axisymmetric mode was employed for the single transducer simulation with lead zirconate titanate, which could be expanded to illustrate 3D output; see Figure 2. The objective was to investigate the acoustic interaction between the transducer surface and air, signal penetration in the air medium, and reflections in the air. The acoustic, electrostatic, and solid mechanics modules from COMSOL Multiphysics were used accordingly. A simple geometry was created for the transducer, representing a rectangle with dimensions of 4.5 mm (width) × 7 mm (height), and the material assigned to it was lead zirconate titanate. The second part of the geometry pertains to the air domain, where the acoustic output propagates and eventually interacts with water droplets to accelerate evaporation. For the basic transducer simulation, the air medium was modelled as a 100 mm radius arc with the centre point coinciding with the transducer’s centre point. Additionally, a 5 mm matching layer was extended along the border. A frequency of 40 kHz was set, and the simulation was executed accordingly.

**Figure 3:**
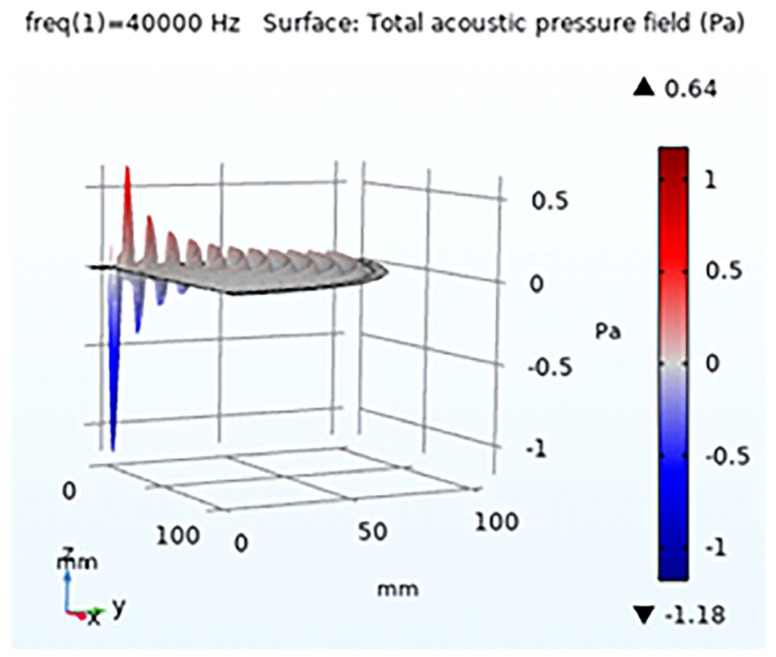
Wave pattern penetration, illustrating crest and troughs. Close to the transducer, high intensity is noted, which gradually decreases as the wave propagates away from the transducer.

**Figure 4:**
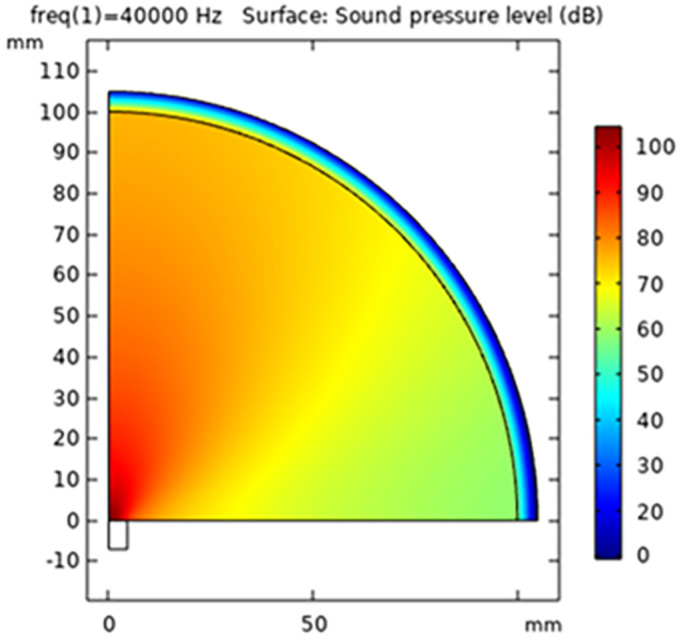
Results from COMSOL finite element analysis (FEA). Colours represent the intensity of the pressure: red indicates maximum pressure, while dark blue is the minimum Sound Pressure Level (SPL) point at the edge.

The simulation results demonstrated a good directivity of waves and significant intensity at very low power input; see Figure 2, Figure 3 and Figure 4. The Sound Pressure Level (SPL) output from the simulation for a single transducer was measured at 101.4 dB when observed at a distance of 30 mm. A formation consisting of multiple transducers was developed and simulated to enhance the output. The arrangement involved 36 transducers positioned in a curved, concave surface in an array configuration, which generated a robust ultrasonic field. This arrangement could be further amplified by mirroring the same configuration. However, in the final design, a constraint was imposed to optimize the distance between the two transducer arrays. While it is known that a closer distance between the arrays would result in higher ultrasonic intensity, it was crucial to ensure that the distance was not too small, as this could impede the introduction of droplets during experimentation. Moreover, the distance should not be smaller than the size of the conduit that is to use to introduce the flowing droplets. This ensured that the practical setup for experimentation remained uncompromised. After arranging two transducers in opposite directions, an optimal distance of 105 mm was determined through simulation, allowing for constructive interaction between the opposing transducer arrays.

**Figure 5:**
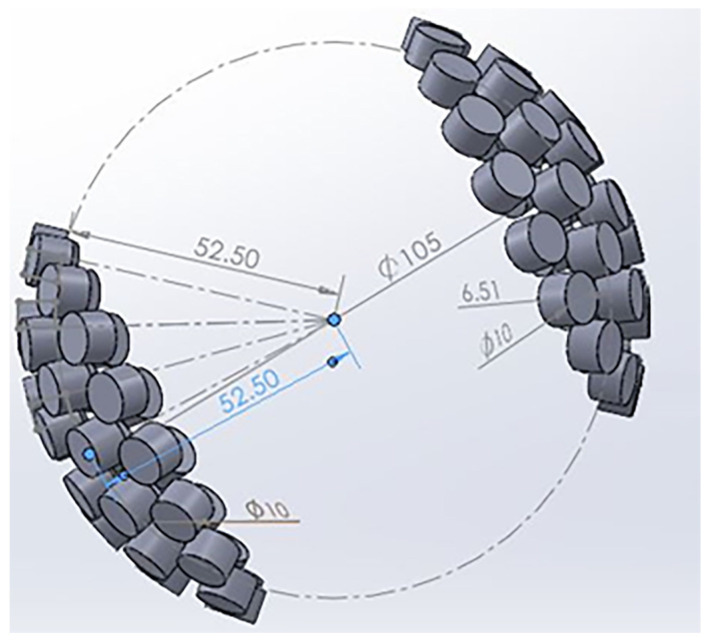
Final CAD design for ultrasonic field generation.

For the final design, a spherical formation was selected. The rationale behind this choice was to ensure that each opposing transducer pair was exactly 105 mm apart, and all transducers would focus on the same point. To initiate the design, a circle with a diameter of 105 mm was generated. The circle was then modified to retain only the curved surface equivalent to the one used in the 36-transducer array design. The base was transformed into a curved surface, with each point situated at a distance of 52.5 mm from a central focal point. Transducers were positioned on the surface, and a mirror image was created at a distance of 105 mm. This arrangement allowed all transducers to focus on the centre point, resulting in a strong ultrasonic intensity at that location; see Figure 5.

**Figure 6:**
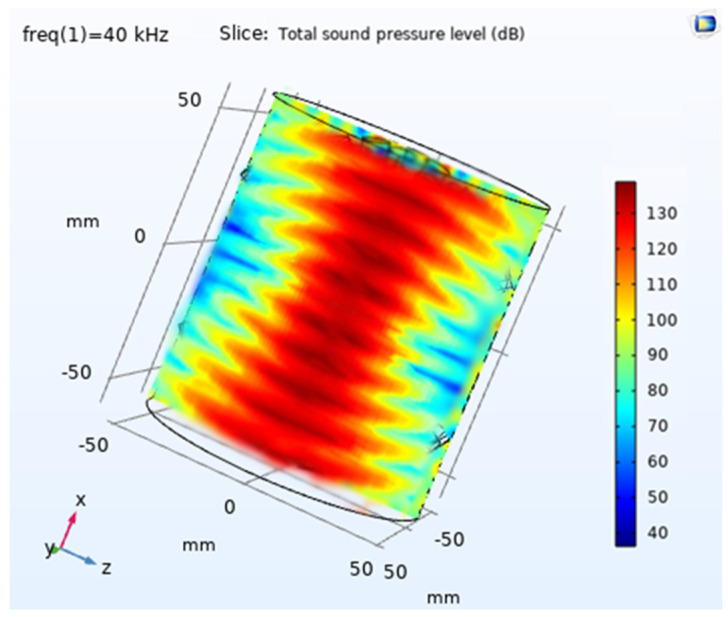
The simulation output of a 72 transducer array, with 36 transducers on each side facing the other set of 36.

The simulation results for the final design demonstrated in Figure 6 that the sound pressure level (SPL) peak achieved at the centre is approximately 140 dB, as observed in the COMSOL simulation. This design successfully fulfils the requirement for a clear path for the droplet to pass through, ensuring a centralized maximum intensity. Additionally, the simulation results confirm that this design generates a highly robust ultrasonic field.

### 2.2. Experimental Setup

**Figure 7:**
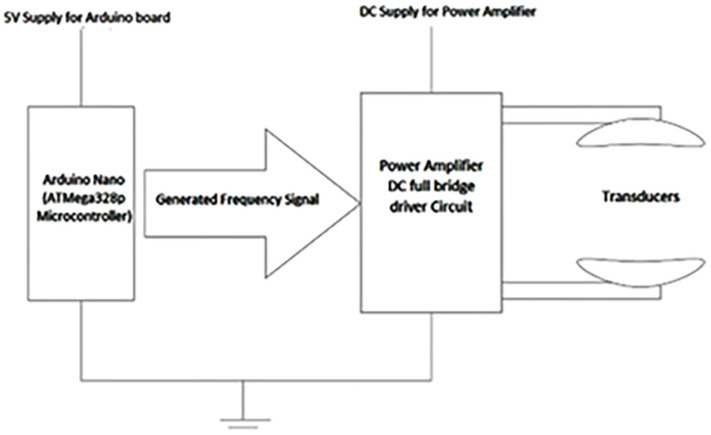
Block diagram for levitator setup.

The Manorshi model MSO-P1040H07TR transducer from Changzou, China, was selected for this study based on capability to withstand electric driving voltage ranging from 5 V to 20 V and mechanical loads, low dielectric loss and high permittivity, a high mechanical quality factor and a high coupling coefficient, and stable properties. A compact and portable setup was devised to activate the transducers, utilizing a battery-powered small signal generator circuit. This circuit comprised a microcontroller and a high-voltage driver. During the design process, careful consideration was given to factors such as the power requirements of the microcontroller and high-voltage driver, the battery capacity and voltage, and the size and weight of the components to ensure ease of portability; see Figure 7. Additionally, safety precautions, including over-current and short-circuit protection, were incorporated into the design to safeguard the circuit and transducers against potential damage.

**Figure 8:**
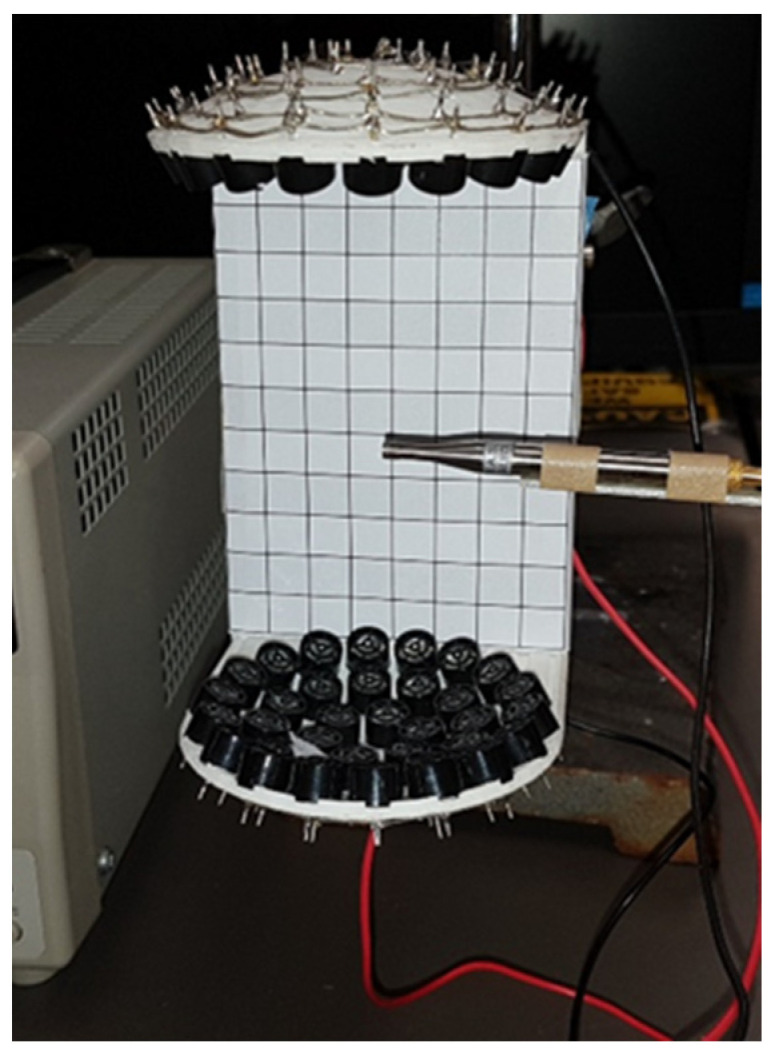
Ultrasonic array connected, and microphone inserted to test SPL.

The transducer was powered up to test the ultrasonic array described in the previous section, and ultrasound intensity was measured using a highly sensitive microphone by GRAS Acoustics, i.e., GRAS 46DP-1 1/8″ LEMO Pressure Standard Microphone Set with a frequency range from 6.5 Hz to 140 KHz, dynamic range measurement capacity of 52 dB to 178 dB and 0.9 mV/Pa sensitivity connected to GRAS 12AL 1-channel CCP Power module with A-weighting filter. The amplifier array testing with GRAS microphone setup is presented in Figure 8.

**Figure 9:**
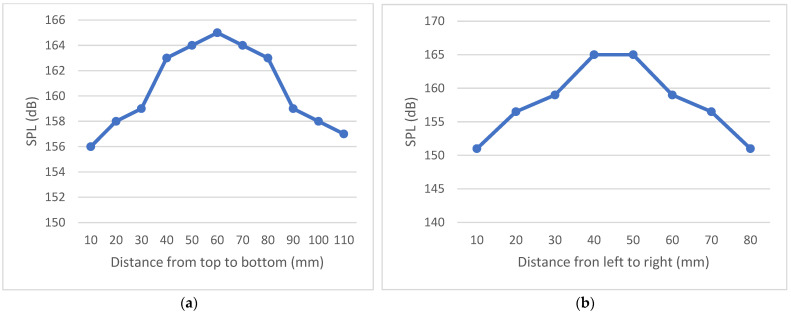
(**a**) SPL values with 10 V supply in centre point from top to bottom (**b**) SPL values with 10 V supply from right to left in the exact centre of XY axis and centre of depth (Z-axis).

The sound pressure level (SPL) was measured from top to bottom, and the highest SPL was observed at the precise centre of the two arrays. When measuring SPL from top to bottom, an increasing pattern of intensity was observed as the microphone approached the centre. Similarly, when the microphone was moved from right to left in the middle row, there was an increasing trend in SPL until it reached the centre. The output in graphical format, presented in Figure 9, indicates that the maximum SPL is expected to occur at the centre location, representing the path with the greatest impact.

### 2.3. Experimental Parameters

To investigate the effect of the second-stage ultrasound field on the evaporation process, a relatively new application of an ultrasound field, the airflow rate, temperature, relative humidity, and total pressure were essential parameters. The important parameters are the conduit size, length (droplet flow path), thermal properties, ultrasound frequency, ultrasound amplitude, and water droplet size distribution.

**Figure 10:**
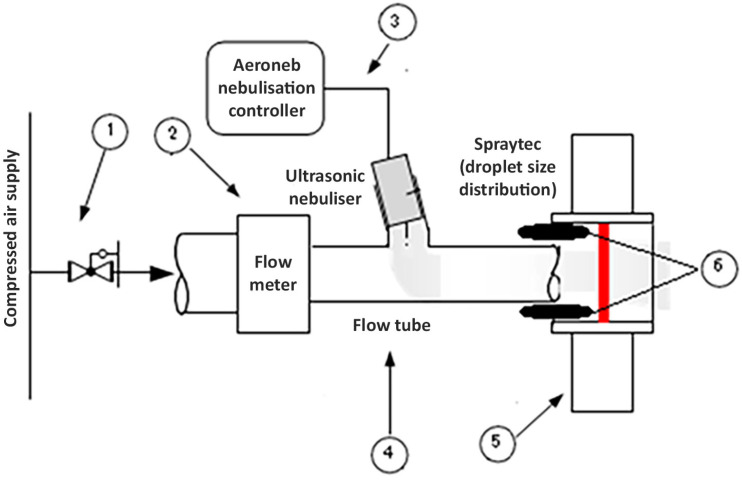
Experiment flow schematic. 1—Air supply regulator, 2—Air-flow meter, 3—Nebuliser controller, 4—Nebulizer reservoir and flow tube, 5—Spraytec Source and detector and 6—Transducers array.

Reducing the droplet size can enhance the evaporation rate, and the pressure applied to the droplet by the ultrasonic field is expected to further improve evaporation rates through diffusion. To maintain consistency, all other parameters were kept constant during the experiment. The flow schematics of the experimental setup are demonstrated in Figure 10 with all labels.

The experiment was conducted at room temperature (22 °C), and the water used for droplet generation was also maintained at room temperature to avoid additional variations in temperatures. The ultrasonic nebulizers used in the experiment did not allow for adjustments in flow rate and droplet size distribution. Hence, these factors remained constant throughout the duration of the experiment. The airflow rate was set to 3 L/min and remained consistent to ensure comparable droplet-to-air ratio measurements in both the ultrasound and non-ultrasound conditions. The optimal frequency for the transducer was determined to be 40 kHz, with a corresponding sound pressure level (SPL) of 165 dB.

### 2.4. Experimentation

**Figure 11:**
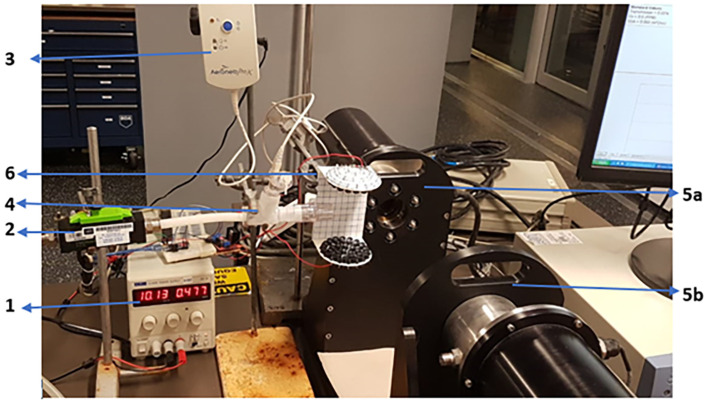
Experimentation setup for flowing droplet evaporation studies. 1—Power supply, 2—Air-flow meter, 3—Nebuliser controller, 4—Nebulizer reservoir and flow tube, 5a and 5b—Spraytec Source and detector and 6—Transducers array. Description of experimental apparatus presented in Appendix B.

To supply clean air for the continuous flow of droplets, an air compressor is employed to supply air for the flow via a filter; to regulate the airflow rate to the system, a needle valve is put at the entry. An airflow meter measures the flow rate. Flow is adjusted using a needle valve as per requirement. Water droplets are produced by an ultrasonic nebulizer, Aeroneb Solo nebulizer and Aeroneb Pro-X Controller, supplied by Aerogen Ltd., Galway, Ireland. The ultrasonic nebulizer is powered by a controller, and distilled water for nebulization is added to a small reservoir inside the nebulizer. The droplets are introduced into a conduit through a T connection that is attached to the conduit. The conduit finally discharges at a Spraytec Laser Diffraction device by Malvern Instruments Ltd., Malvern, UK, for minute particle size measurements, to measure the droplet size distribution of flowing droplets. The experimentation setup is presented in Figure 11.

## 3. Results

**Figure 12:**
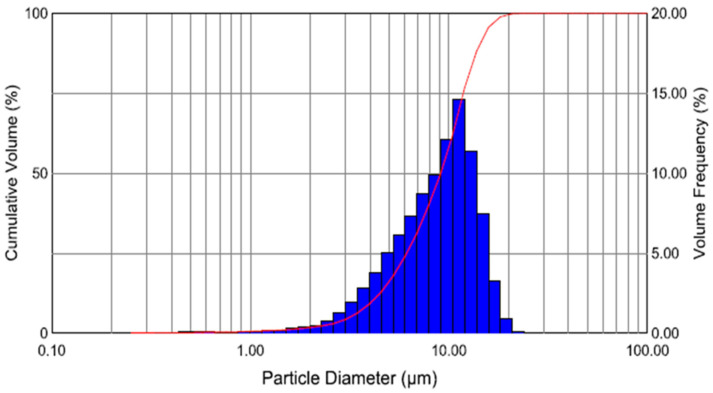
Droplet size distribution: % volume versus droplet diameter in absence of second stage ultrasonic field.

**Figure 13:**
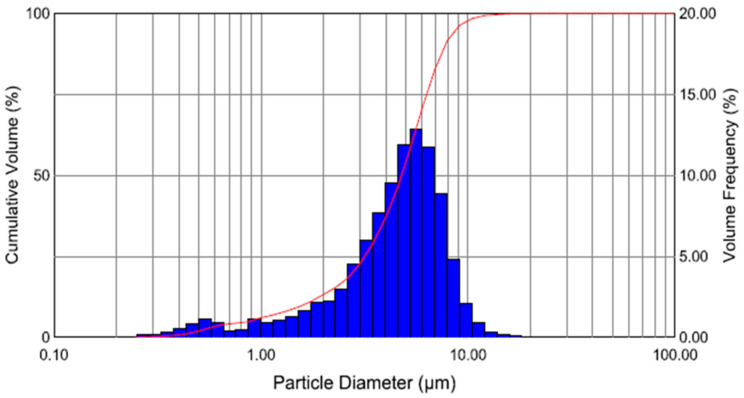
Droplet size distribution: % volume versus droplet diameter in presence of second stage ultrasonic field.

The above two histograms, Figure 12 and Figure 13, are output from the Malvern laser droplet particle analyser software v3.20. The vertical, blue-coloured bars represent “bins” of droplet sizes, which can be read off the abscissa.

The volume frequency (as a percent of the total volume) can be read off the axis on the right side. Examining Figure 12, the droplet distribution in the absence of a second stage ultrasonic field, we find that the droplets in the 10-micron bin represent approximately 11% of the total volume, and approximately 48% of the droplet volume is over 10 microns. There were no droplets smaller than a micron and a negligible number of droplets with a size larger than a micron. In Figure 13, when the second stage ultrasonic field is switched on and stable, only 3% of droplets with a size of 10 microns were recorded compared to 11% when the ultrasonic field was absent. In the same manner, the composition of droplets over 10 microns in size in total volume reduces to approximately 6% from 48% measured before the ultrasonic field. One percent of the total droplet volume measured one micron in diameter, while droplets less than one micron in size were also recorded with the ultrasonic field. Overall, more than 60% of the volume of the droplets are about six microns or less. The red, s-shaped curve shows the cumulative volume across the size bins.

## 4. Discussion

The impact of the high-intensity ultrasonic field on moving droplets in the air is obvious. The objective is an alternative design of the humidifier for lung therapeutic devices, and all such devices require continuous vapour flow. The experimentation setup for flowing droplet interaction and recording impact is detailed with typical results. The study requires the determination of accurate droplet size, as it is most important for concluding this study. For generating droplets to pass through the ultrasonic field, the nebulizer was used, which provides a wide range of statistically distributed droplets in sizes of a few micrometers in diameter, and after the droplets experience the ultrasonic field, their size alters. With and without a second stage ultrasound field, the droplets will have size differences to be counted and differentiated, from submicron to a few microns. The studies taken into consideration were imaging (microscopy and high-speed video cameras) and laser light scattering techniques. The high-speed camera recorded a two-dimensional image of the droplets from a single, front-facing direction, which limited its ability to capture droplet overlap or the full three-dimensional structure of the droplets. This limitation was particularly noticeable when two different droplets partially overlapped from the front view, as it was difficult to accurately distinguish between them, resulting in larger droplets being observed; also, if smaller droplets hide behind them, the large droplet could not be counted.

In the laser diffraction technique, particles are passed through a laser, and the scattering pattern is measured by several detectors. The Spraytec laser diffraction device is supplied by Malvern Instruments Ltd. in England. The Spraytec device requires a specific density of droplets to give an accurate reading; for flowing droplets, there will be enough density that can be used in the laser light scattering technique, which works on the principles of the spatial and flux techniques. The spatial technique instantly samples many droplets in a given volume. This is a number density-weighted technique, and Malvern Instruments is a benchmark manufacturer.

The flux or temporal technique samples and counts individual droplets passing through the sampling volume (established by the cross-section of two beams of laser light) in a given time interval and is a number flux-weighted technique.

Many researchers confirmed the preciseness, repeatability, and measurement stability of the Malvern system. Based on the feedback of the aforementioned researchers, the Malvern system has opted for this study. Figure 12 and Figure 13 are output from the Malvern laser droplet particle analyser software. The vertical, blue-coloured bars represent “bins” of droplet sizes, which can be read off the abscissa. The volume frequency (as a percent of the total volume) can be read off the axis on the right side. In the droplet distribution in the absence of a second stage ultrasonic field, we find that the droplets in the 10-micron bin represent approximately 11% of the total volume, and approximately 48% of the droplet’s volume is over 10 microns. There were no droplets smaller than a micron and a negligible number of droplets with a size larger than a micron. In Figure 13, when the second stage ultrasonic field was switched on and stable, only 3% of droplets with a size of 10 microns were recorded compared to 11% when the ultrasonic field was absent. In the same manner, the composition of droplets over 10 microns in size in total volume reduces to approximately 6% from 48% measured before the ultrasonic field. One percent of the total droplet volume measured one micron in diameter, while droplets less than one micron in size were also recorded with the ultrasonic field. Overall, more than 60% of the volume of the droplets are about six microns or less. The red, s-shaped curve shows the cumulative volume across the size bins.

**Figure 14:**
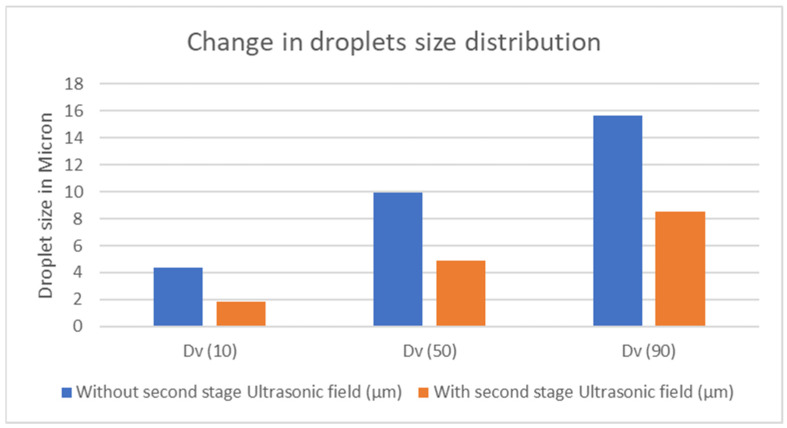
Comparison of droplet distributions in percent.

The levitator-style second stage ultrasonic field creates a strong acoustic field and significantly amplifies the variations in droplet distribution compared to its absence, and particularly affects the larger droplets, as evident by a shift in the histogram peak presented in Figure 14.

## 5. Conclusions

The study investigates a new ultrasonic transducer-based design of a humidifier for lung therapeutic devices that offers more control, is more power-efficient, and is practical in size for use with CPAP and other lung therapeutic devices. Ten percent of the total measured droplets were found to be under or equal to 4.37 µm. After passing through the ultrasonic field, 10% of the droplets measured 1.85 µm or less. In the absence of the second stage ultrasonic field, 50% and 90% of the total droplets measured under or equal to 9.93 µm and 15.67 µm, respectively. However, with the ultrasound field in the second stage, the droplets reduced to 4.88 µm and 8.54 µm for 50% and 90% of measurements, respectively. This implies an approximately 45% reduction in droplet sizes when measured for the overall 90% of the total, while for the smaller droplets, it is found that there was over a 55% reduction. The study focused on a multistage ultrasonic humidification approach using an ultrasonic levitator design. The successful design of the levitator provided a high-intensity ultrasonic field that was capable of levitating and disintegrating droplets, and furthermore resulted in a decreased droplet size distribution as the droplets flowed through the field.

The implication of this research is that imposing an ultrasound field on evaporating droplets can have a significant effect on the droplets. It certainly improves evaporation rates, and this could have a beneficial application for medical breathing devices and related applications.

## Data Availability

The original contributions presented in the study are included in the article, further inquiries can be directed to the corresponding author.

## Appendix A

Parameters B << A details included in Appendix A.

$$\frac{dr}{dt}=\frac{1}{\rho r}\;\left\{\frac{DM_{w}}{R}\left[\;\frac{x_{w}P_{w}^{vap}\left(T_{s}\right)e^{\frac{2oV_{m}}{rRT_{s}}}}{T_{s}}-\frac{(RH)P_{w}^{vap}\left(T_{\infty }\right)}{T_{\infty }}\right]+\frac{k_{TH}RT_{\infty }T_{s}}{M_{w}\lambda_{w}^{2}\;}\left[1-\frac{X_{w}P_{w}^{vap}\left(T_{s}\right)e^{\frac{2oV_{m}}{rRT_{s}}}}{P_{w}^{vap}\left(T_{\infty }\right)}\right]\;\right\}$$

The exponential term in the equation can be simplified to the

$$e^{\frac{2oV_{m}}{rRT}}≅1+\frac{2oV_{m}}{rRT}$$

this gives:

$$\frac{dr}{dt}=\frac{1}{\rho r}\;\left\{\frac{DM_{w}}{R}\left[\;\frac{x_{w}P_{w}^{vap}\left(T_{s}\right)}{T_{s}}-\frac{(RH)P_{w}^{vap}\left(T_{\infty }\right)}{T_{\infty }}\right]+\frac{k_{TH}RT_{\infty }T_{s}}{M_{w}\lambda_{w}^{2}\;}\left[1-\frac{X_{w}P_{w}^{vap}\left(T_{s}\right)e^{\frac{2oV_{m}}{rRT}}}{P_{w}^{vap}\left(T_{\infty }\right)}\right]\;\right\}+$$

$$\frac{X_{w}P_{w}^{vap}\left(T_{s}\right)}{\rho r^{2}}\frac{2oV_{m}}{RT_{s}}\;\left\{\frac{DM_{w}}{R}-\frac{k_{TH}RT_{\infty }T_{s}}{M_{w}\lambda_{w}^{2}\;}\;\right\}$$

$$A=\frac{1}{\rho }\;\left\{\frac{DM_{w}}{R}\left[\;\frac{x_{w}P_{w}^{vap}\left(T_{s}\right)}{T_{s}}-\frac{(RH)P_{w}^{vap}\left(T_{\infty }\right)}{T_{\infty }}\right]+\frac{k_{TH}RT_{\infty }T_{s}}{M_{w}\lambda_{w}^{2}\;}\left[1-\frac{X_{w}P_{w}^{vap}\left(T_{s}\right)e^{\frac{2oV_{m}}{rRT}}}{P_{w}^{vap}\left(T_{\infty }\right)}\right]\;\right\}$$

$$B=\frac{X_{w}P_{w}^{vap}\left(T_{s}\right)}{\rho }\frac{2oV_{m}}{RT_{s}}\;\left\{\frac{DM_{w}}{R}-\frac{k_{TH}RT_{\infty }T_{s}}{M_{w}\lambda_{w}^{2}\;}\;\right\}$$

The differential equation can be express as:

$$\frac{dr}{dt}=\frac{A}{r}+\frac{B}{r^{2}}$$

Integrating the equation with the initial condition at *t* = 0, *r* = *r*_0_,

$$\frac{A\left(r^{2}-r_{0}^{2}\right)-2B\left(r-r_{0}\right)}{2A^{2}}+\frac{B^{2}}{A^{3}}\ln\left(\frac{Ar+B}{Ar_{0}+B}\;\right)=t$$

This equation can now present the evaporation rate dependent on the radius. It can be solved numerically and if *B* ≪ *A*, then the result is:

$$r^{2}-r_{0}^{2}=\frac{2}{\rho }\;\left\{\frac{DM_{w}}{R}\left[\;\frac{x_{w}P_{w}^{vap}\left(T_{s}\right)}{T_{s}}-\frac{\left(RH\right)P_{w}^{vap}\left(T_{\infty }\right)}{T_{\infty }}\right]+\frac{k_{TH}RT_{\infty }T_{s}}{M_{w}\lambda_{w}^{2}\;}\left[1-\frac{X_{w}P_{w}^{vap}\left(T_{s}\right)}{P_{w}^{vap}\left(T_{\infty }\right)}\right]\;\right\}\;t$$

Based on this theoretical model, if a droplet is spherical in shape, and placed in a standard room environment as follows,

- ρ (Density of water) = 1000 kg/m^3^
- D (Diffusion coefficient of water vapour in air) = 2.3 × 10^−5^ m^2^/s
- R (Gas constant) = 8.31 J/(mol-K)
- T_s_ (Temperature at the surface of the droplet) = 298 K
- T_∞_ (room temperature) = 293 K
- RH (Relative humidity) = 50%
- $k_{TH}$ (Thermal conductivity of air) = 0.026 W/(m K)
- λ_w_ (Latent heat of vaporisation) = 2.55 × 10^6^ W/(m K)
- M_w_ (Molecular weight of water) = 0.018 kg/mol
- P_0_ Initial pressure inside the droplet = 101,325 Pa (assuming standard atmospheric pressure)
- x_w_ (Concentration of water vapour at the surface of the droplet) = 0.0256 kg/m^3^ calculated using the saturation vapour pressure of water at Ts
- $P_{w}^{vap}\left(T_{s}\right)$ (Water vapour pressure at surface temperature) = 3163.6 Pa (from a steam table at 298 K)
- $P_{w}^{vap}\left(T_{\infty }\right)$ (Water vapour pressure at room temperature) = 2439.7 Pa (from a steam table at 298 K)

From the above calculations, it takes 124 s for a 2 mm diameter droplet to evaporate completely. However, if the same quantity of liquid is broken into 10 equal parts of 0.2 mm each, the time for complete evaporation reduces to 12.4 s. This indicates that reducing the size of the droplet by 10 times accelerates the evaporation process by 10 times while keeping all other conditions constant. This study also suggests that droplets can be manipulated or disintegrated when subjected to a high-intensity ultrasonic field, and the overall increase in ultrasonic pressure can play a role in the process.

## Appendix B

**Table A1:** Description of experimental apparatus.

Item	Description	Details
1	air supply, filter regulator	Existing air supply. B72G Filter/Regulator, F72G 5 micron filter. Norgren Ltd., Auckland, New Zealand.
2	air flow meter	CMOSens^®^ EM1 Mass flow meter for gases. Model No. EM1NV4R0V_1A suitable for 0–200 L/min. Sensirion AG, Stäfa, Switzerland
3	water nebulisation transducer and controller	Aeroneb Solo nebuliser, Aeroneb Pro-X Controller. Aerogen Ltd., Ireland
4	flow tube	25.4 × 22 mm polycarbonate tube. Mulford Engineering Plastics, Auckland, New Zealand.
5	droplet size distribution measurement	Spraytec97 laser diffraction droplet sizer, RTSizer software. v3.20 Malvern Instruments Ltd., UK
6	ultrasonic transducers array	Manorshi model MSO-P1040H07TR, Ghangzou, China transducers used to design array
